# Oral dextrose gel for hypoglycemia in a well-baby nursery: a baby-friendly initiative

**DOI:** 10.1038/s41372-024-02114-y

**Published:** 2024-09-14

**Authors:** Mansi Batra, Kelechi Ikeri, Michelle Blake, Genevieve Mantell, Ramachandra Bhat, Michael Zayek

**Affiliations:** 1 Department of Pediatrics, Division of Neonatology, University of South Alabama, Mobile, AL USA; 2 Department of Nursing, USA Children’s and Women’s Hospital, Mobile, AL USA; 3 Quality Care Coordinator. Gulf Coast Total Care, Mobile, AL USA; 4 Department of Neonatology, KLE Academy of Higher Education and Research, Jawaharlal Nehru Medical College, Belagavi, India

**Keywords:** Outcomes research, Risk factors

## Abstract

**Objectives:**

To assess the impact of oral dextrose gel (ODG) treatment on NICU admission rates for hypoglycemic infants in a well-baby nursery.

**Study design:**

We retrospectively compared newborn infants at risk for hypoglycemia born during the intervention period (*n* = 3775) with historical controls (*n* = 655). We also compared the rates of the primary outcome (NICU admission) and secondary outcomes (exclusive breastfeeding and hospital costs) between the two periods.

**Results:**

Following the implementation of ODG supplementation, the NICU admissions rates dropped from 4% to 2%, *p* < 0.05, for at-risk infants and from 15% to 7%, *p* < 0.05, for hypoglycemic infants in the baseline and intervention periods, respectively, with an adjusted OR (95% CI) of 0.39 (0.24–0.64), *p* < 0.001. Additionally, the ODG protocol sustained rates of exclusive breastfeeding in contrast to the institutional protocol.

**Conclusion:**

The adoption of an ODG protocol fosters a more nurturing and baby-friendly environment through reduced NICU transfers, support for exclusive breastfeeding, and decreased hospital costs.

## Introduction

Based on available evidence from several published studies [1–3], a number of well-baby nurseries have adopted the use of oral dextrose gel (ODG) for the initial management of hypoglycemia.

These studies have highlighted its advantages, such as reducing the separation of mothers and infants, decreasing admissions to the Neonatal Intensive Care Unit (NICU), and promoting exclusive breastfeeding in newborns with hypoglycemia. Furthermore, the use of ODG was considered safe, with no adverse neurosensory outcomes observed in infants at 2 years, 4.5 years, and 9 years of age [2, 4, 5].

Surprisingly, more recent studies investigating the potential advantages of ODG therapy have questioned its main benefits. The primary goal of ODG therapy is to prevent the separation of newborns from their mothers by avoiding NICU admissions for intravenous therapies. A systematic review of two randomized control trials (RCTs) raised doubts about the effectiveness of ODG in reducing the need for intravenous hypoglycemia treatment [6].

Additionally, a recent multicenter RCT that examined the prophylactic use of a single dose of ODG in at-risk infants observed a decrease in the incidence of hypoglycemic episodes without a corresponding reduction in NICU admissions [7]. In fact, the overall impact of ODG on the absolute risk reduction (ARR) of hypoglycemia was not as robust as previously reported in studies with similar cohorts (with ARRs of 5% and 18%, respectively). In that study [7], the failure to demonstrate a reduction in NICU admissions with ODG therapy was attributed to multiple factors. These factors included variable thresholds across centers for escalation to NICU admission for further hypoglycemia management, inadequate ODG dosing with single-dose regimens, and supplementation with bovine milk-derived newborn formula. In February 2017, our institution implemented an ODG-based protocol for the initial management of hypoglycemia in the well-baby nursery, aligning with the published reports on the use of ODG supplementation [1, 2] and the 2011 AAP guidelines for neonatal hypoglycemia [8]. However, one potential barrier to a successful ODG implementation at our institution could be the greater utilization of bovine milk-derived newborn formula compared to centers that demonstrated successful ODG use [1, 9]. Given these concerns and the conflicting results from recent studies evaluating the benefits of ODG therapy, we sought to ascertain the utility of this treatment in reducing NICU admission rates for hypoglycemia at our institution. The study included infants admitted to the well-baby nursery who exhibited low blood glucose (BG) levels requiring intervention. We, therefore, conducted a retrospective review of these infants who received ODG therapy following the implementation of this protocol.

## Methods

### Research ethics and consent

This single-center retrospective cohort study was conducted at the University of South Alabama in Mobile, Alabama, with approval from the institutional review board’s ethics and research committee (Reference 23–374). The committee ensured that the study adhered to all relevant guidelines and regulations. Given the study design, obtaining informed consent was not feasible.

### Study design

We included healthy newborn infants who met the following criteria: gestational age of ≥ 34 weeks, birth weight >2000 g, born from April 2016 through March 2023, and considered to be at high risk for transitional hypoglycemia. We excluded infants born in 2017 between February and December from the study due to data unavailability caused by our institution’s electronic medical record system switch from Cribnote (Grand Rounds ^TM^ Software, LLC. Philadelphia, PA) to Cerner (Oracle Health, Kansas City, KS) that year. The implementation of the ODG protocol began in February 2017

**Fig. 1:**
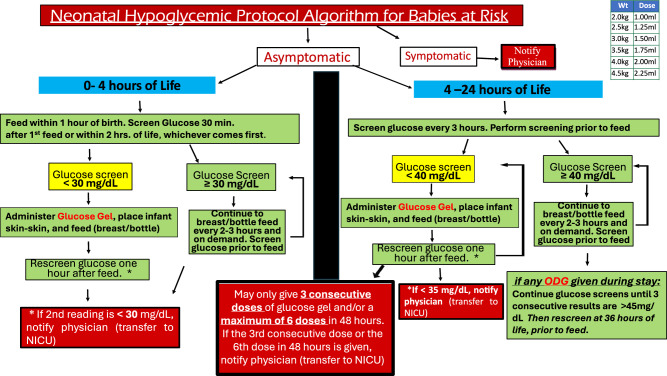
Protocol for management of infants at risk for hypoglycemia during the first 24 h of life during the intervention period. This algorithm is adapted from the 2011 AAP guidelines, with modifications incorporating the administration of 40% oral dextrose gel (ODG, 0.5 ml/kg) for hypoglycemia. An asterisk (*) denotes critical blood glucose values at which point immediate transfer to the neonatal intensive care unit (NICU) is recommended, rather than a repeat glucose screen.

Consequently, we categorized the infants into two groups: the baseline group, consisting of historical control infants, born before the ODG implementation (April 2016 to January 2017) and managed according to an institutional protocol; the intervention group, including infants born from January 2018 through March 2023, during a period during which we implemented an ODG protocol which was incorporated into the 2011 AAP guidelines (Fig. 1).

**Fig. 2:**
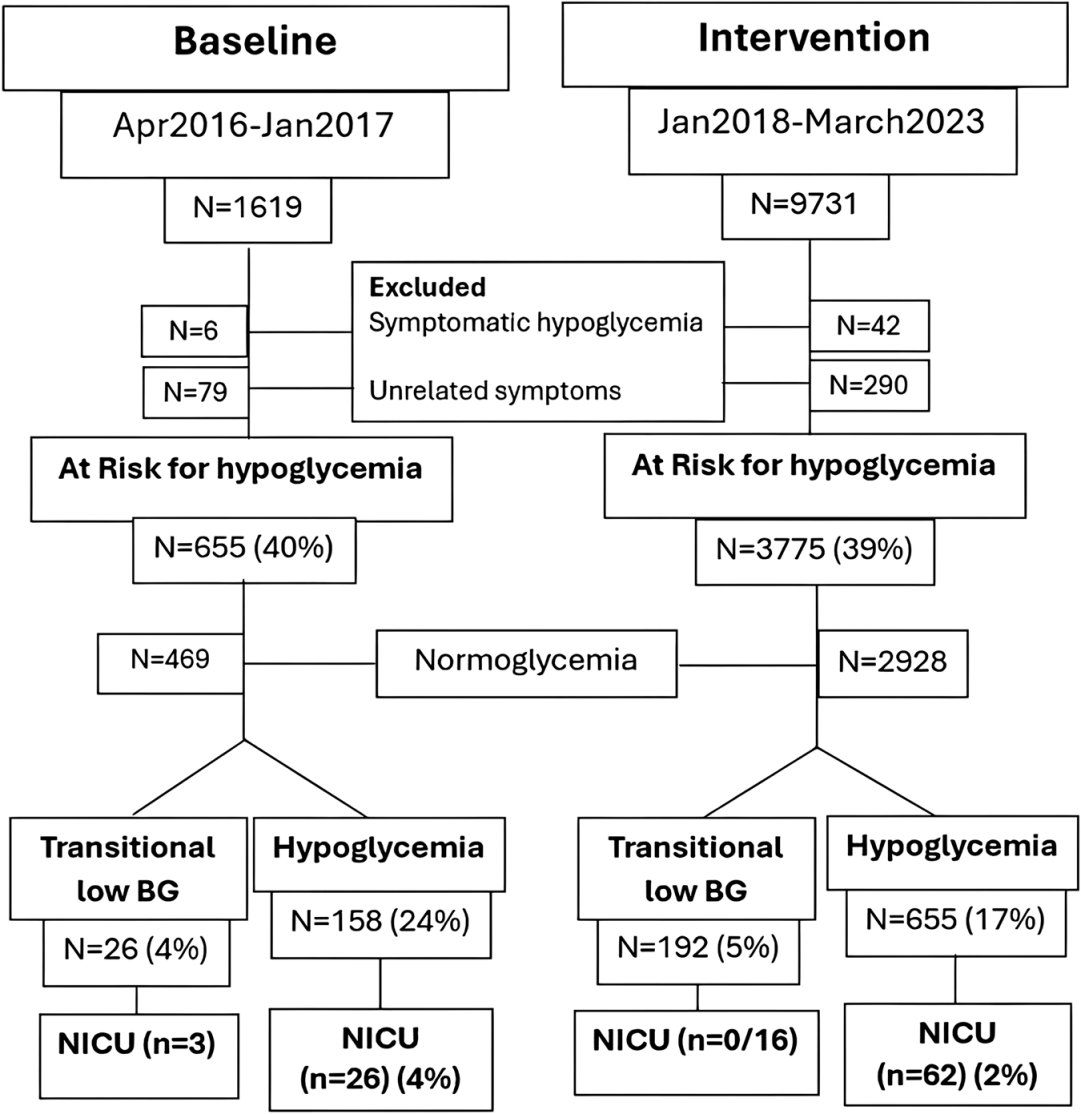
Flow diagram of healthy term and late preterm infants admitted to the well-baby nursery. This figure illustrates the incidence of transitional low blood glucose and hypoglycemia among infants at risk and subsequent management during the baseline and intervention periods. * *p* < 0.05, significantly lower rates of hypoglycemia in the intervention group compared to baseline.

We excluded infants with hypoglycemia that was associated with other unrelated symptoms warranting NICU transfer such as tachypnea, alarming abdominal signs and symptoms, or neonatal abstinence syndrome (Fig. 2).

### Protocols and interventions

We defined infants at risk for hypoglycemia as any infant who received a point-of-care (POC) BG test in the newborn nursery (NBN). The definition of hypoglycemia for infants >4 h of age was similar to the 2011 AAP guidelines (BG levels < 40 mg/dl) and remained unchanged between the two periods. However, during the first 4 h of life, transitionally low BG levels (30–39 mg/dl) were managed differently between the two periods. In the intervention period, their management followed the 2011 AAP guidelines, considering these levels physiologic and normal, whereas during the baseline period, they were considered abnormal. In the intervention period, BG levels <30 mg/dl were considered hypoglycemia in the first 4 h of age.

During the baseline period, the operational threshold for hypoglycemia was a BG level of <40 mg/dl within the first 24 h. Asymptomatic infants with hypoglycemia received their initial feeding through either breastfeeding or bottle-feeding. The criteria for transferring infants to the NICU included any subsequently confirmed BG level <35 mg/dL during the first 24 h of life, or three consecutive borderline BG levels between 35–39 mg/dL.

During the intervention period, hypoglycemic infants received an initial supplementation of 0.5 ml/kg of 40% oral dextrose gel (Glutose-15 40% ODG, Perrigo, Minneapolis, MN) sublingually. This was followed by gently massaging the gel onto the buccal mucosa before feeding. However, in February 2021, the unit switched from using the Glutose-15 formulation to Sweet Cheeks (40% ODG, manufactured by DandleLion Medical in Danbury, CT) due to concerns regarding parabens [10, 11]. The criteria for transferring infants to the NICU were any subsequent BG level <30 mg/dL within the first 4 h of life, any BG level <35 mg/dL from 4 to 24 h of life [12], three consecutive doses of ODG, or more than six cumulative doses of ODG (Fig. 1). Infants transferred to the NICU for hypoglycemia after 24 h of age (BG levels <45 mg/dl) [12] were excluded from this study, with 3 cases occurring during the baseline period and 7 during the intervention period.

### Glucose measurements

Infants identified as at risk for hypoglycemia underwent serial BG level screening tests as recommended by the 2011 AAP guidelines during both the baseline and intervention periods. BG levels were screened by obtaining a small blood sample from a heel stick using a POC testing device (Accu-Chek Inform II, Roche Diagnostics, Abbott Park, IL). We ensured that all POC testing devices were used in accordance with the operator’s manual. Any POC BG levels <50 mg/dL were subsequently confirmed using the glucose oxidase method (RapidLab blood gas analyzer, Siemens Diagnostics, Deerfield, IL), conveniently located near the nursery. NICU transfer was based on confirmed BG levels, measured using the glucose oxidase method.

### Data variables

In this study, we collected demographic data, including maternal diabetes, gestational age (GA), and birth weight (BW), to identify at-risk infants: infants born to diabetic mothers (IDM), preterm infants (<37 weeks GA), small for gestation (SGA, <10th percentile), and large for gestation (LGA, >90th percentile) based on Fenton’s intrauterine growth chart criteria [13]. Additionally, some infants were screened for hypoglycemia despite having no risk factors but exhibited clinical concerns such as excessive weight loss and/or breastfeeding insufficiency. We retrieved data on feeding practices during hospital stays, categorizing infants into two subgroups: exclusively breastfeeding or breastfeeding with exposure to bovine-based milk. Additionally, we compiled data on major daily hospital expenses, primarily consisting of room and professional charges for infants in both the NBN and the NICU. We also identified reasons for NICU transfers, specifically those transferred solely for hypoglycemia management.

### Data collection

We collected relevant clinical, laboratory, and treatment data for infants in our study. For infants born during the baseline period, data were acquired from individual charts created in Cribnote and later scanned into Cerner. For infants born after December 2017, data was retrieved from generated Cerner reports.

### Outcome measures

The primary outcome was the rate of NICU admission among the at-risk infant cohort of the current study. The NICU admission was defined as any asymptomatic newborn admitted to the NBN for routine care who required BG monitoring, developed low BG levels necessitating interventions, and was subsequently transferred to the NICU due to failure to achieve normoglycemia.

Secondary outcomes included NICU admissions strictly for hypoglycemia as defined by AAP, NICU admissions specifically for borderline low BG levels during the transition period (BG 30–39 mg/dl, <4 h of life), feeding practices at the time of hospital discharge, such as exclusive breastfeeding, breastfeeding in combination with bovine-based milk formula, the cost of hospital care, and recurrent hypoglycemia. Recurrent hypoglycemia was defined as the occurrence of another episode of low blood sugar within 48 h after birth, following an initial successful treatment. All of these outcomes were compared between the two periods. Furthermore, the study cohort was stratified based on the timing and type of low BG levels (transitional low BG levels or hypoglycemia) and classified into etiology-based risk-group subtypes for comparing the primary outcome between the two periods.

### Statistical analyses

Statistical differences between the baseline and intervention groups were analyzed using Pearson’s chi-square test for categorical data and a two-sample Wilcoxon rank-sum (Mann- Whitney) test for continuous variables not normally distributed. We used multivariate logistic regression to adjust for any significant association between the study periods (exposure variable) and primary outcome or any secondary outcomes (dependent variable) by including baseline characteristic variables that are different between the groups in univariate analysis (*p* < 0.05). All baseline characteristics that were included in the multivariate regression analysis were sequentially excluded either for *p* > 0.2 or when Bayesian Information Criteria of the depleted model were less than the prior model by an absolute value more than 2 [14]. Measures of association were expressed as odds ratio (OR) with 95% confidence intervals (CI). All statistical analyses were conducted using Stata 13 (StataCorp LP, Lakeway Drive, College Station, TX).

## Results

Out of the 11350 charts examined, 48 infants were excluded due to symptomatic hypoglycemia. Figure 2 illustrates that 4430 (39%) infants were at risk for hypoglycemia. Hypoglycemia rates were significantly different between periods: 24% in the baseline period versus 17% in the intervention period (*p* < 0.05).

Table 1 compares characteristics of at-risk infants between the two periods. This table indicates that hypoglycemia rates among at-risk infants decreased during the intervention period, despite a higher proportion of infants born to diabetic mothers (48% vs. 43%, *p* = 0.036) and preterm births (29% vs. 25%, *p* = 0.031). Additionally, fewer at-risk infants without risk factors were tested mainly due to clinical concerns during the intervention compared to baseline (4% vs. 10%, respectively, *p* = 0.021). Among these, fewer showed low BG levels during the intervention period (4% vs. 13%, *p* < 0.001).

**Table 1:** Characteristics of at-risk infants for hypoglycemia.

	Baseline Period			Intervention Period		
	At-Risk	Transitional Low BG	Hypoglycemia	At-Risk	Transitional Low BG	Hypoglycemia
	*N* = 655	*N* = 26	*N* = 158	*N* = 3775	*N* = 192	*N* = 655
GA (weeks)	39 (37–39)	37 (36–39)	37 (36–39)	38 (36–39)	37 (36–39)	37 (36–39)
BW (grams)	2916 (2620–3541)	2985 (2550–3682)	2974 (2546–3662)	3152 (2555–3605)	2880 (2500–3465)	2905 (2497–3501)
Hospital days	3 (2–4)	3 (2–5)	3 (2–5)	3 (2–4)	3 (2–4)	3 (2–4)
NBN stay	3 (2–4)	3 (2–4)	3 (2–4)	3 (2–4)	3 (2–4)	3 (2–4)
NICU stay		5 (2–10)	4 (3–6)		6 (4–11)	6 (4–11)^c^
BW < 2500 g	112 (17)	3 (11)	32 (20)	832 (22)^c^	45 (23)	165 (25)
BW > 4500 g	6 (1)	0	3 (2)	53 (1)	0	11 (2)
BW < 10th	157 (24)	2 (8)	17 (11)	850 (22)	37 (19)	134 (20)^c^
BW > 90th	119 (18)	6 (22)	34 (21)	583 (15)	18 (9)^c^	109 (17)
Late preterm	161 (25)	6 (23)	57 (36)	1083 (29)^c^	65 (34)	208 (32)
34 weeks	30 (5)	0	11 (7)	172 (5)	16 (8)	41 (6)
35 weeks	54 (8)	3 (11)	24 (15)	319 (8)	20 (10)	58 (9)
36 weeks	77 (12)	3 (11)	22 (14)	592 (16)^c^	29 (15)	109 (17)
IDM	284 (43)	13 (46)	66 (42)	1804 (48)^c^	69 (36)	298 (45)
Others^a^	64 (10)	6 (23)	21 (13)	172 (4)^c^	24 (12)	28 (4)^c^
Cow-Based formula^b^	287 (44)	9 (35)	67 (43)	1891 (50)^c^	91 (47)	296 (45)

Continuous variables are represented as median (IQR), categorical variables as *n* (%).

Hypoglycemia was defined as per 2011 AAP guidelines. Hypoglycemic infants in the intervention period received oral dextrose gel. Transitional low BG levels were considered normal and physiologic during intervention period as per 2011 AAP and abnormal during the baseline period as per institutional protocol.

^a^Others: infants who have no risk factor for hypoglycemia but had clinical concerns.

IDM: infant of diabetic mother, GA: gestational age, BW: birth weight.

^b^Number (%) of infants who were exclusively fed cow-based formula.

^c^*P* < 0.05, intervention vs baseline between similar subgroups.

More importantly, a significant drop in hypoglycemia rates was associated with more exclusive formula feeding during the intervention period, particularly among at-risk normoglycemic infants (50% vs. 44%, *p* = 0.008), especially in infants of diabetic mothers (44% vs. 36%, *p* = 0.018). Exclusive formula feeding rates among hypoglycemic infants of diabetic mothers were similar (60% vs. 59%, *p* = 0.91).

During the intervention, the proportion of small-for-gestational-age (SGA) hypoglycemic infants increased from 11% to 20% (*p* = 0.005), while proportions of infants of diabetic mothers and preterm births remained similar.

**Fig. 3:**
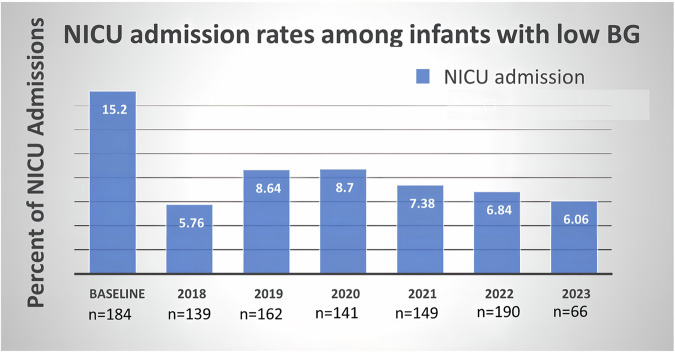
Rates of NICU admission during the baseline period and each year of the intervention period. A linear regression analysis revealed significantly lower rates throughout all years of the intervention period compared to the baseline. Of note, the data for year 2023 covers only the first 3 months, from January through March.

The primary outcome, transfer to the NICU due to low BG, decreased significantly during the intervention. The transfer rate for at-risk infants dropped from 4% to 2%, and for those with low BG levels from 16% to 7% (Table 2). With an 83% study power, the intervention was significantly associated with lower NICU admissions (OR 0.39, 95% CI 0.24–0.64, *p* < 0.001), after adjusting for various factors. Infants of diabetic mothers and those with clinical concerns were also less likely to be admitted to the NICU (OR 0.51, 95% CI 0.31–0.80 and OR 0.16, 95% CI 0.04–0.7, respectively). Figure 3 depicts a notable decrease in NICU admission rates in the years subsequent to the implementation of the ODG supplementation protocol. Throughout the intervention period, all years showed significantly lower rates compared to the baseline, as confirmed by linear regression analysis.

**Table 2:** NICU Admission Rates are categorized according to risk factors.

	Baseline	Intervention	*p* values
	Apr2016-Jan2017	Jan2018-March2023	
At Risk for hypoglycemia	29/655 (4)	62/3775 (2)^c^	<0.001
Any low BG^a^	29/186 (16)	62/847 (7)^c^	<0.001
Hypoglycemia	26/158 (16)	62/655 (9)^c^	0.011
Transitional low BG	3/26 (11)	0^a^	<0.001
Hypoglycemia and LGA	6/18 (18)	13/109 (12)	0.391
Hypoglycemia and SGA	1/17 (6)	11/134 (8)	0.738
Hypoglycemia and Late Preterm	13/57 (23)	27/208 (13)	0.066
Hypoglycemia and 34 weeks	2/11 (18)	6/41 (14)	0.772
Hypoglycemia and 35 weeks	9/24 (37)	4/58 (7)^c^	0.001
Hypoglycemia and 36 weeks	2/22 (9)	17/109 (16)	0.429
Hypoglycemia and IDM	11/66 (17)	20/298 (7)^c^	0.009
Hypoglycemia and Others^b^	1/21 (5)	0	0.323

Variables represent the ratio of number of infants admitted to NICU/Total number of infants (%).

*IDM* infant of diabetic mother, *SGA* small for gestational age, *LGA* large for gestational age.

^a^Any Low BG (blood glucose) includes infants exhibiting transitional low BG or hypoglycemia.

^b^Others: infants who have no risk factor for hypoglycemia but had clinical concerns.

^c^*p* < 0.05, statistical significance between baseline and intervention periods.

During the intervention period, 192 infants had transitional low BG levels (30–39 mg/dL) in the first 4 h of life. Sixteen had subsequent low levels, but none required NICU transfer, per 2011 AAP guidelines. In contrast, during the baseline period, 3 infants (11%) were transferred to the NICU due to being SGA, LGA, or poor feeding with significant weight loss. Implementing the AAP guidelines led to a significant drop in NICU transfers from 11% to 0%, *p* < 0.001, even when adjusted for risk factors and feeding modality.

Of 655 hypoglycemic infants receiving ODG supplementation during the intervention, NICU admissions were lower (9% vs. 16%, *p* = 0.011). Table 2 shows this decrease was significant among preterm infants, those born to diabetic mothers, and those with clinical concerns. After adjusting for these risk factors, the association remained significant (OR 0.49, 95% CI 0.29–0.81, *p* = 0.005).

During the intervention period, breastfeeding rates for at-risk infants declined from 56% to 50% (*p* = 0.003), but exclusive breastfeeding rates for those with low BG improved from 21% to 34% (*p* < 0.001). This improvement was noted in both transitional and hypoglycemia groups (Table 3). Adjusted analyses confirmed the significance (OR 1.64, 95% CI 1.07–2.52, *p* = 0.023).

**Table 3:** Breastfeeding Rates in Infants at Risk for hypoglycemia.

	Baseline			Intervention		
	Transitional	Hypoglycemia	Normoglycemia	Transitional	Hypoglycemia	Normoglycemia
	*N* = 26	*N* = 158	*N* = 469	*N* = 192	*N* = 655	*N* = 2928
Any Mother’s Milk	17 (65)	91 (58)	260 (55)^a^	101 (53)	359 (55)	1424 (49)
Exclusive Mother Milk	4 (15)^a^	34 (22)^a^	150 (31)	71 (36)	221 (34)	935 (32)
Exclusive: Any Ratio	0.23^a^	0.37^a^	0.58	0.7	0.62	0.65

^a^*p* < 0.05, baseline vs. intervention within the same subgroup of glycemia: transitional, hypoglycemia, or normoglycemia. Variables are represented as *n* (%).

Furthermore, we calculated hospital expenses for healthy newborns with hypoglycemia, considering those who remained in the NBN and those who were transferred to the NICU.

Cost analysis showed a NICU stay added $5,334 per patient compared to the NBN (Table 4). Reducing NICU transfers from 16% to 7% prevented 76 transfers, saving $405,384 over 5.25 years ($77,216 per year).

**Table 4:** Cost per hospitalization.

	NICU charges			NBN charges		
	Room	Professional	Total	Room	Professional	Total
Admit day ($)	2116	1013	3129	400	257	657
Discharge day ($)	1204	210	1414	400	210	610
In Between days ($)	1204	347	1551	400	122	522
Hospital stays (days)	4 (3–6)	4 (3–6)	4 (3–6)	3 (2–4)	3 (2–4)	3 (2–4)
Hospital costs ($)	5728 (4524–8136)	1917 (1570–2611)	7645 (6094–10747)	1600 (1200–1600)	711 (589–833)	2311 (1789–2833)

The included costs assume the lowest estimate scenario: only first day in NICU and all subsequent days in lower-level special care in NICU.

Values are represented as median (interquartile range) or as a single value for daily charge.

There was no increase in recurrent hypoglycemic episodes during the intervention. Regarding hyperglycemia [15], only one instance (0.1%) occurred during the baseline period (BG of 176 mg/dL), compared to three cases (0.5%) during the intervention among infants treated with ODG, each experiencing a single episode (126, 127, and 140 mg/dL). A small proportion of preterm infants received prenatal steroids (16% during the baseline period vs. 10% during the intervention, *p* = 0.011), but this had no significant impact on the incidence of hypoglycemia (*p* = 0.417) or the primary outcome.

## Discussion

In this retrospective study, our findings revealed that incorporating ODG in the protocol based on the AAP guidelines for managing neonatal hypoglycemia resulted in a decrease in NICU admissions, the preservation of exclusive breastfeeding, and a reduction in hospital costs. This decline in the primary outcome of NICU admissions was observed in infants with both transitional low BG levels and hypoglycemia. Among infants with hypoglycemia, the decline in NICU admissions was mainly seen among 35-week infants and infants of diabetic mothers. Furthermore, episodes of recurrent hypoglycemia or any occurrence of hyperglycemia were not increased.

While ODG supplementation is effective in reducing NICU admissions, the magnitude of its effect, as measured by the Absolute Risk Reduction (ARR), varies across published reports [1, 7, 9]. Notably, the Number Needed to Treat (NNT) in Gupta et al. [9] was much smaller than in our study, indicating that one NICU admission was prevented for every three cases of ODG use in their research, whereas our NNT was one for every twelve cases of ODG use.

This difference could be attributed to their primarily exclusive breastfeeding population. These variations in protocols and feeding practices may help explain the challenges encountered in achieving a significant reduction in NICU admissions in recent studies using ODG [7].

Exploring other efficient alternatives to ODG therapy for hypoglycemia may mitigate the confounding effects of feeding practices in enhancing BG levels. For instance, the use of a single intramuscular injection of glucagon has been shown to be effective in reducing NICU admissions for hypoglycemic infants [16, 17]. However, this therapy is invasive. A single dose of glucagon does not cover later occurrences of hypoglycemia and raises concerns as it may lead to a rapid increase in BG levels. In fact, hyperglycemia or the rapid correction of hypoglycemia, has been associated with neurosensory impairment [18, 19].

Distinguishing the sole contribution of ODG supplementation in reducing NICU admissions from the adherence to a protocol-defined criteria presents a challenge. For instance, the implementation of a hypoglycemia protocol based on AAP recommendations, which emphasized skin-to-skin care without the use of dextrose gel, significantly decreased unnecessary NICU admissions, from 17% to 3% in infants at risk for hypoglycemia [20]. In fact, NICU admission rates for at-risk infants at our institution during the baseline period were similarly low at 4%. Further, accepting an operational threshold for hypoglycemia at 30 mg/dl in the first 4 h also contributed to the reduction in NICU transfer. The additional incorporation of ODG further reduced our NICU admission rates. Indeed, a randomized control study which compared ODG within a similar protocol to ours against a placebo showed a similar reduction in NICU admission rates, dropping from 40% to 10% [9]. This convergence of results underscores the complexity of attributing the reduction in NICU admissions solely to ODG supplementation while emphasizing the importance of consistently integrating ODG within specific guidelines. The combination of hypoglycemia protocol and ODG could have cumulatively contributed to the reduction in NICU admissions. Therefore, we recommend incorporating ODG into the unit protocol for optimal results.

ODG supplementation has gained significant parental acceptance due to its ability to support exclusive breastfeeding and prevent separation from the mother [21]. In addition, the approach of serial monitoring of the infant in the NBN with intervention such as OGD therapy or NICU transfer only when indicated has been proven to be safe [2, 4, 5]. Other advantages, such as a reduction in hospital expenses, have also been described. This reduction in healthcare costs allows for the reallocation of funds to other critical areas within the healthcare ecosystem. Legitimate concerns have been raised regarding the inactive components present in dextrose gel, particularly the inclusion of methyl and propyl parabens [10, 11]. This harmful exposure of neonates is not limited to ODG, which is primarily used in the well-baby nursery, but extends to other medications administered in the NICU, including acetaminophen, furosemide, and multivitamins, particularly biotin. Thus, care in NICU serves as a potential source of exposure to parabens [22]. To address these concerns, we transitioned to a paraben-free, artificial flavor-free, and dye-free gel formulation in February 2021. This 40% glucose gel formulation is conveniently packaged in small vials to facilitate precise dosing, eliminating the need for pharmacist preparation, as required with Glutose-15. The key advantage of this new formulation is the absence of unnecessary additives, such as preservatives, artificial flavors, and dyes, while also reducing NICU admissions and the associated potential exposure to other commonly used medications [22].

The main limitation of this study is its retrospective design, which inherently limits control over variables and introduces potential biases. We noted a variation in hypoglycemia rates among at-risk infants between the study periods. One possible factor contributing to this variation is the increased proportion of infants exposed to formula feeding during the intervention period. Previous research by Harris et al. [23] found that glucose response is greater after formula feeding compared to expressed human milk, which may explain the observed decline in hypoglycemia rates. However, while this change in feeding practice might have reduced hypoglycemia rates among at-risk infants, it should not have influenced the response of infants with low BG levels during the implementation of the ODG protocol.

In conclusion, our study highlights that ODG is safe and beneficial when incorporated into a protocol based on recommendations from the AAP. The benefits of this practice include a reduction in NICU transfers, facilitation of the vital bond between newborns and their mothers by preventing separation, preservation of exclusive breastfeeding, and a reduction in hospital costs. The adoption of an oral dextrose gel protocol contributes to the development of a more nurturing and baby-friendly environment. However, further prospective studies are warranted to validate and build upon our findings.

## Data Availability

The datasets generated during and/or analyzed during the current study are available from the corresponding author upon reasonable request.
